# Policies and resources for strengthening of emergency and critical care services in the context of the global COVID-19 pandemic in Kenya

**DOI:** 10.1371/journal.pgph.0000483

**Published:** 2023-07-03

**Authors:** Jacquie Narotso Oliwa, Rosanna Jeffries Mazhar, George Serem, Karima Khalid, Patrick Amoth, Helen Kiarie, Osman Warfa, Carl Otto Schell, Tim Baker, Mike English, Jacob Mcknight

**Affiliations:** 1 Department of Health Systems & Research Ethics, KEMRI-Wellcome Trust Research Programme, Nairobi, Kenya; 2 Department of Paediatrics & Child Health, University of Nairobi, Nairobi, Kenya; 3 Nuffield Department of Medicine, Health Systems Collaborative, University of Oxford, Oxford, United Kingdom; 4 Department of Emergency Medicine, Muhimbili University of Health and Allied Sciences, Dar es Salaam, United Republic of Tanzania; 5 Ifakara Health Institute, Dar es Salaam, United Republic of Tanzania; 6 Office of the Director General, Ministry of Health, Nairobi, Kenya; 7 Division of Monitoring and Evaluation, Ministry of Health, Nairobi, Kenya; 8 Department of Global Public Health, Karolinska Institutet, Stockholm, Sweden; 9 Centre for Clinical Research Sörmland, Uppsala University, Eskilstuna, Sweden; 10 Department of Internal Medicine, Nyköping Hospital, Nyköping, Sweden; 11 Department of Clinical Research, London School of Hygiene & Tropical Medicine, London, United Kingdom; Universidade de São Paulo: Universidade de Sao Paulo, BRAZIL

## Abstract

Critical illnesses cause several million deaths annually, with many of these occurring in low-resource settings like Kenya. Great efforts have been made worldwide to scale up critical care to reduce deaths from COVID-19. Lower income countries with fragile health systems may not have had sufficient resources to upscale their critical care. We aimed to review how efforts to strengthen emergency and critical care were operationalised during the pandemic in Kenya to point towards how future emergencies should be approached. This was an exploratory study that involved document reviews, and discussions with key stakeholders (donors, international agencies, professional associations, government actors), during the first year of the pandemic in Kenya. Our findings suggest that pre-pandemic health services for the critically ill in Kenya were sparse and unable to meet rising demand, with major limitations noted in human resources and infrastructure. The pandemic response saw galvanised action by the Government of Kenya and other agencies to mobilise resources (approximately USD 218 million). Earlier efforts were largely directed towards advanced critical care but since the human resource gap could not be reduced immediately, a lot of equipment remained unused. We also note that despite strong policies on what resources should be available, the reality on the ground was that there were often critical shortages. While emergency response mechanisms are not conducive to addressing long-term health system issues, the pandemic increased global recognition of the need to fund care for the critically ill. Limited resources may be best prioritised towards a public health approach with focus on provision of relatively basic, lower cost essential emergency and critical care (EECC) that can potentially save the most lives amongst critically ill patients.

## Introduction

Critical illness is thought to cause several million deaths globally each year with many of these deaths occurring in low-resourced settings like Kenya [1,2]. The global incidence of critical illness before the COVID-19 pandemic is unknown, but was crudely estimated at 30–45 million per year, and in 2016, approximately 8.6 million deaths occurred in lower income countries from causes that ‘should not occur in the presence of timely and effective healthcare’ [2]. Improving the quality and availability of critical care is essential if this burden is to be reduced. In high income countries (HICs), there are approximately 5–30 intensive care unit (ICU) beds/100,000, compared with 0.1–2.5 ICU beds/100,000 in lower income settings [2,3]. Intensive care in HICs often involves expensive equipment, high quality diagnostic support and large numbers of highly trained staff. In lower resourced settings, the situation is very different with: low staff-patient ratios (shortages in nursing staff often leading to relatives caring for the critically ill); poor pre-hospital care accompanied by inefficient health seeking behaviour and late illness presentations; lack of proper hospital systems for managing critically ill patients; and finally, the cost of intensive care is prohibitive even amongst the higher income earners in low resource settings [2]. All these factors likely contribute to the higher mortality observed amongst the critically ill in these settings.

The COVID-19 pandemic became an unprecedented critical care crisis, with a severe shortage in critical care capacity, even in HICs. The significant limitations of critical care in low-resource settings were also immediately exposed. A recent report showed that mortality in patients with COVID-19 treated in ICUs or high-care units was significantly higher in Africa (close to 50% compared with the global average of 31.5%) [4]. Delays in admissions due to insufficient critical care resources was reported as one of the drivers of mortality in this study.

Earlier response efforts globally were focussed on scaling up highly advanced ICU technological capabilities to mitigate deaths from the pandemic worldwide. It has become increasingly clear however, that there is an important role for more basic forms of critical care such as rapid identification of critical states and administration of oxygen and fluids, and a growing acknowledgement that effective, early intervention with basic, often neglected steps could potentially save many lives, especially in low-income settings [5,6]. As COVID-19 lacked definitive treatment, critical care (support for vital organs) has been the primary means of reducing mortality.

Essential Emergency and Critical Care (EECC) is a new concept developed by a consensus process that encompasses all the basic, low-cost actions required by critically ill patients and the system wide requirements for their provision [7,8] (see The EECC framework in S1 Fig). The COVID-19 crisis provided a unique opportunity for improving care and generating knowledge about feasible, critical care systems strengthening that could potentially save the lives of many critically ill patients in the long run. While lower- and middle-income countries (LMICs) may aspire to global best practice for the clinical management of critically ill patients, it is crucial for the global health community to reflect on where new resources might best be allocated [9]. Low-resource settings may not have sufficient strength in their health systems to install, effectively use, and maintain high level critical care for all critically ill patients. The much-needed influx of capacity-building funding may be best aimed at ensuring the provision of the care that can potentially save lives amongst critically ill patients.

We explored how efforts to strengthen care were operationalised in the context of the COVID-19 pandemic in Kenya. Specifically, we describe which policies, strategies and coordination mechanisms were adopted, the underlying assumptions and motivations for these, how they were implemented including at sub-national levels and what resources were allocated. We paid special attention to i) upgrading or re-purposing facilities; ii) purchase and distribution of equipment or other technologies; iii) supply of new consumables like personal protective equipment; iv) provision of training to health workers; and v) recruitment of additional health workers. This broad analysis allows us to reflect on how effective current policies are for guiding action and to point towards areas for potential improvement.

## Methods

### Study setting

This work focused on Kenya and the first year the COVID 19 pandemic, from March 2020 to April 2021. Kenya has a surface area of 580,367 km^2^ and a population of approximately 47 million [10]. It has a young population, with >70% younger than 30 years of age. Economically, Kenya is classed as a low-middle income country with a with a gross national income (GNI) per capita of USD1600. Almost 20% of the population live on less than USD2 a day [11].

**Fig 1:**
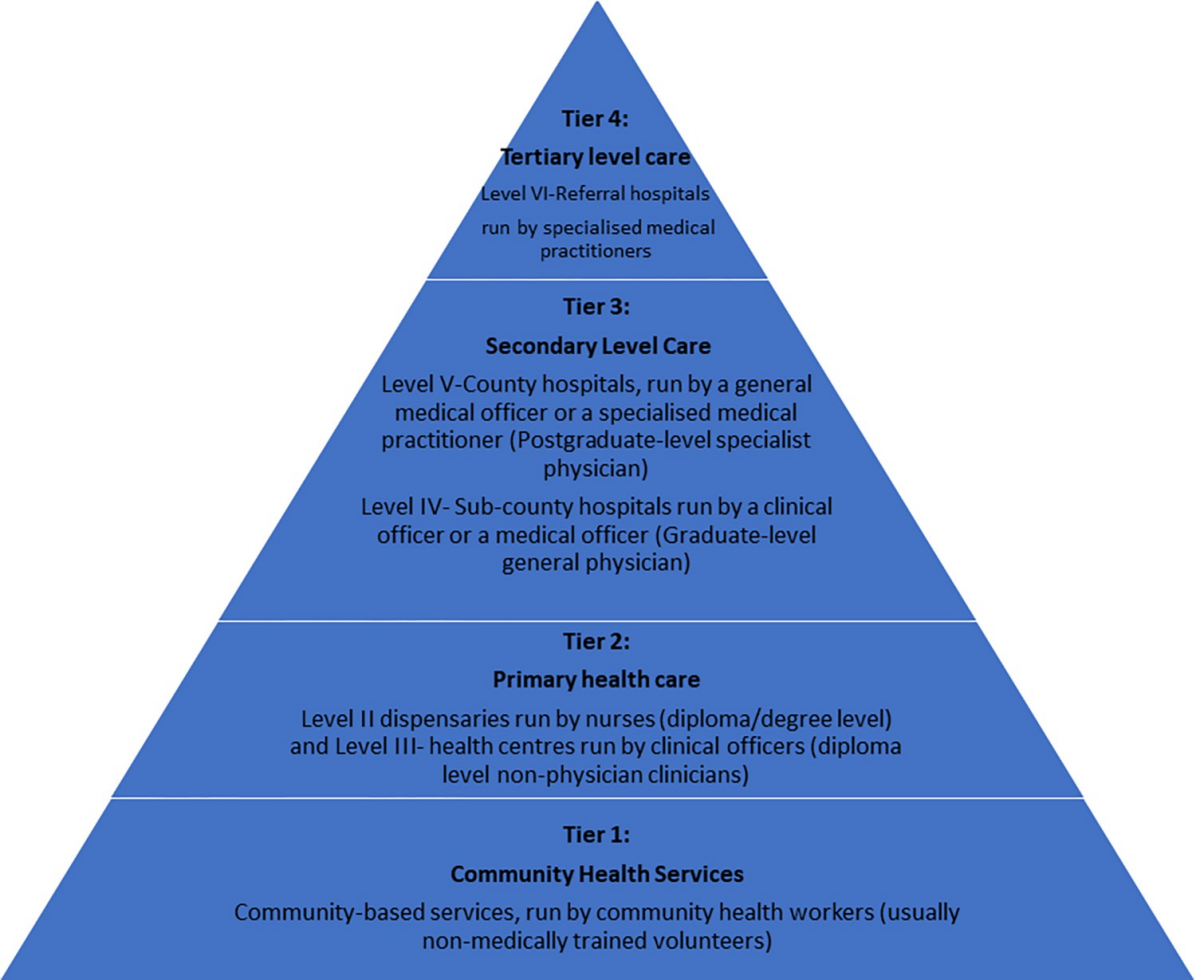
Structure of Kenya Health care delivery.

Clinical services in the public sector in Kenya are organised in four tiers since devolution (see Fig 1), with upward referral from the community towards the more advanced tertiary level care found at the national referral hospitals. Critically ill patients will likely be seen first wherever they present at all levels of care and may be managed at that level or referred upwards after stabilisation, depending on the expertise and care required. Intensive Care Units (ICUs) will mainly be found in Level V and Level VI hospitals (in Tiers 3 and 4) in Kenya. There is a policy expectation that there is one Tier 3 facility per 100,000 population capable of providing services similar to EECC, while ICU services are much fewer [12].

Kenya’s available educational opportunities for healthcare workers to specialise in anaesthesia, intensive care and emergency medicine are presented in Table 1, along with figures on available human resources. It should also be noted that these aggregates mask unequal distribution, given the tendency for specialised staff to be concentrated at larger referral centres in urban centres of Nairobi, Kisumu and Mombasa.

**Table 1:** Human resources and formal educational opportunities: Anaesthesia, intensive care and emergency medicine in Kenya from available reports by 2021.

	Kenya
**Population**	46,050,000
**Human Resources for Health: general**	• 8682 physicians [13,14] (1.9: 10,000 population) • 31,896 nurses and midwives (2015) [15]
**Anaesthesia and intensive care**	• 202 physician anaesthesia providers (0.44: 100,000 population) • 108 nurse anaesthesia providers [13,14] • 234 critical care nurses (2015) [15]
**Emergency medicine**	• 2 specialist physicians in emergency medicine (2018/2019) [16] • 35 accident and emergency nurses (2015) [15]
**Accredited Training: emergency medicine, anaesthesia and critical care**	• 2 universities offer specialist training in anaesthesia for doctors ^a^ • No standardized curriculum or licensure of any Emergency Medicine training for medical doctors in Kenya [17]. • Several institutes offer accredited 12-month higher diplomas to train as a Kenya Registered Nurse Anaesthetist ((KRNA))^b^ and a Kenya Registered Critical Care Nurse ((KRCCN)^c^ • One institute offers a Registered Emergency Medicine and Critical Care Clinical Officers (ECCCO) Diploma ^d^ for clinical officers • One institute offers an 18-month Diploma in Primary Emergency Care for medical officers, through the College of Emergency Medicine of South Africa^e^ • Two institutes offer 12-month advanced diplomas for nurses to qualify as a Kenya Registered Accident & Emergency Nurse ((KRAEN))^f^ • One institute offers a Registered Emergency Medicine and Critical Care Clinical Officers (ECCCO) Diploma ^g^ for clinical officers

^a^ University of Nairobi and Aga Khan University Hospital.

^b^ KMTC Main campus, KMTC Kisumu, KMTC Kisii, Cecily McDonnell School of Nursing, KNH, Moi Teaching and Referral Hospital, Machakos Medical Training College, Kijabe, and Amref International University.

^c^ Nairobi Women’s Hospital College, Cecily McDonnell School of Nursing, Catherine McAuley Nursing School.

^d^ AIC Kijabe College of Health Sciences, since 2015.

^e^ The Aga Khan University, Nairobi, since 2017.

^f^ Cicely McDonell College of Health Sciences, MMCT St Joseph Kenya.

^g^ AIC Kijabe College of Health Sciences, since 2015.

**Fig 2:**
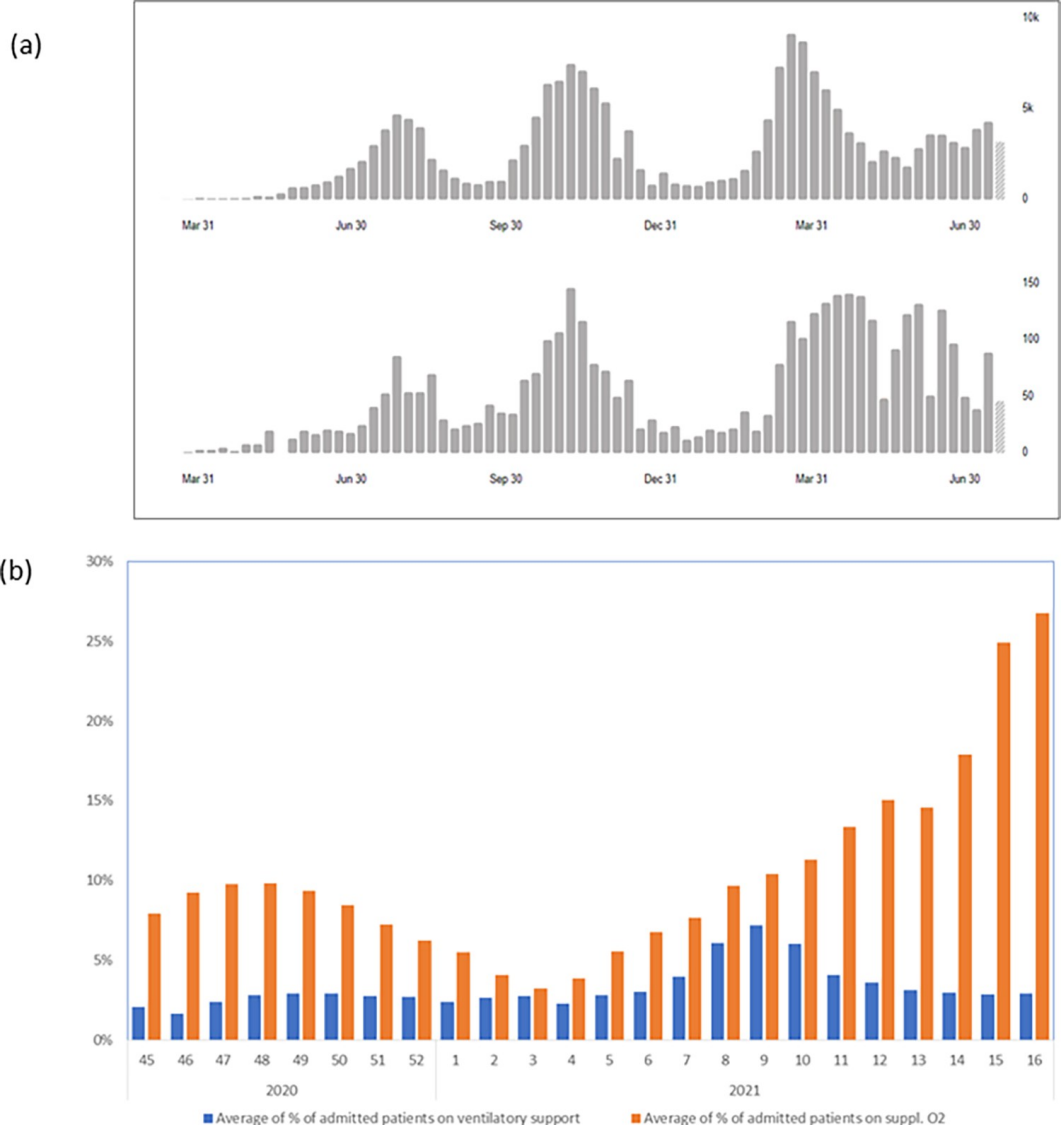
(a) Kenya’s Covid 19 situation overview, with the first graph showing confirmed cases over time and the second graph showing COVID 19 related deaths. Source: WHO Covid 19 regional data https://covid19.who.int/region/afro/country/ke [18]. (b) Kenya’s Covid situation showing proportion of admitted patients on oxygen and ventilatory support over the waves.

Kenya confirmed its first COVID-19 case on March 13^th^ 2020, and as of 22^nd^ April 2021, had reported 154,392 confirmed COVID-19 cases, and 2560 deaths, with three waves peaking in August 2020, November 2020, and April 2021, seen in Fig 2(A) [18]. The third wave was characterised by increased ICU admissions and the emergence of an oxygen crisis, as seen in Fig 2(B) which illustrates the proportion of admitted Covid patients who needed to be on oxygen and ventilatory support.

The Kenyan government implemented strong containment measures including nationwide curfews, lockdowns and bans on movement in response to fluctuating positivity rates. The response was, however, marked by two controversies: strikes by health care workers from November 2020-February 2021 due to grievances related to: a) delayed payment of salaries; b) insufficient personal protective equipment (PPE); c) lack of insurance and compensation; and d) accusations of misappropriation of funds, which led to an inquiry by parliament in October 2020.

### Study design

This was an exploratory qualitative study that involved document reviews and discussions with key stakeholders reflecting on the first year of the pandemic, with the aim of reviewing how scale up of care for critically ill patients was operationalised in the context of the COVID-19 pandemic in Kenya.

### Study process, sampling and data collection

Data collection was between November 2020 and April 2021 and involved two phases:

- **Desk review:** Government and donor reports, literature, website and media sources were reviewed and scanned for information pertaining to the research topic. These documents were identified through key word searches using generic search engines (google) and specialised and academic databases (google scholar); and through a snowballing approach based on documents mentioned during stakeholder discussions and through attending Every Breath Counts Coalition [19] weekly calls. While all efforts were made to ensure comprehensiveness in the desk review, given the breadth of documentation available, the ever-evolving situation and the multiplicity of sources, some reference materials may have been unintentionally excluded.
- **Discussions with stakeholders:** these were held with stakeholders considered to be key informants (donors, international agencies, professional associations, government actors) identified through the desk review process and based on the research team’s country-specific knowledge (see S1 Table with details of stakeholder discussions). We reached out to stakeholders from 20 organisations mapped out in the emergency and critical care space and they were invited via email to share their knowledge of the unfolding COVID response during telephone or online calls structured to help elaborate the emerging picture of the response. Participation was entirely voluntary, and we got responses from 15 organisations and spoke to 19 participants in total. With the participants’ permission, discussions were recorded for note-taking purposes only and any recordings were deleted on completion of notetaking. Due to the sensitive nature of the information shared, participants opted not to be quoted verbatim to protect their identities. Several attempts were made to schedule discussions with stakeholders, and for those who did not respond, attempts were made to retrieve information through publicly available resources.

### Data analysis

RJM conducted independent coding of notes from discussions with participants in NVivo vs 12, to identify and develop key themes, validate and expand on the findings from the desk review and draw key findings relevant to this project. A grounded approach was used to guide the analysis. Descriptive open codes were used initially, and these were subsequently grouped into broad emerging themes. RJM, JNO, JM, ME, COS and TB met weekly to reflect on emerging issues and to develop consensus on the key findings.

### Credibility and trustworthiness

A diverse group of stakeholders was identified for this study, to provide as much depth of understanding as possible on the resources being mobilised and potentially made available to strengthen clinical care in Kenya. Regular meetings of the study team that included a senior social scientist, implementation scientists, clinicians with experience of clinical settings in Kenya, and critical care specialists was key to making sense of the findings and ensuring reflexivity. We also shared findings to stakeholders and to the Ministry of Health in Kenya, to check the validity of our findings. The Consolidated criteria for Reporting Qualitative Studies (COREQ) checklist [20] was used for further quality assurance.

### Ethics

The Kenya Medical Research Institute (KEMRI) Scientific and Ethical Review Committee (SERU Number 4085) and London School of Hygiene and Tropical Medicine (REF 22 866) approved the study. Participants were given a brief introduction to who the researchers were, reasons for doing the research and what the study entailed. They were reassured that their confidentiality would be maintained by omitting personal identifiers and that there would no verbatim quotes used for this report. They gave consent for discussions and for audio-recording via email. Data were stored electronically in password-protected laptops only accessed by the research team.

Additional information regarding the ethical, cultural, and scientific considerations specific to inclusivity in global research is provided in S1 Checklist.

## Results

We present findings from document reviews and discussions with key stakeholders in this section, reflecting on changes during the pandemic and activities of both the government and non-state actors in the following areas i) Human resources/Personnel; ii) Training; iii) Services and equipment; iv) Policy efforts; v) Funding.

### 1. Human resources/personnel to care for the critically ill

While the COVID-19 pandemic saw renewed global awareness of the need to resource health systems to manage severely ill patients, many of the pandemic challenges reflected persistent, long-standing human resource for health (HRH), training and structural constraints in Kenya. As seen in Table 1 for instance, Kenya only has 202 physician anaesthesiologists and 234 critical care nurses to serve a population of approximately 47 million, which falls below the recommended ratios in Table 2. As a result of shortages in trained specialists, there is a strong reliance on generalist (non-specialist) and junior doctors, non-physician clinical officers and nurses to manage critically ill patients, under the supervision of the very few on call-specialists.

**Table 2:** Ratio of the core health workers to patient per shift in the general isolation ward and critical care units in Kenya in 2020 [21].

Cadre/specialisation	Requirement in general isolation ward	Requirement in Critical Care Units
**Anaesthesiologist/ Emergency physician/ Family physician (with training)**	-	Ratio of 1:6 patients
**Physician**	-	Ratio of 1:6 patients
**Medical Officer**	Ratio of 1:10 patients	Ratio of 1:6 patients
**Clinical Pharmacist**	Ratio of 1:50 patients	Ratio of 1:50 patients
**Clinical Officer**	Ratio of 1:10 patients	Ratio of 1:6 patients
**Nurse**	Ratio of 1:5 patients	Ratio of 1:1 patient
**Chest physiotherapist**	Ratio of 1:10 patients	Ratio of 1:6 patients
**Nutritionist**	2 per isolation centre	Ratio of 1:10 patients
**Medical lab technologist**	Ratio of 1:20 patients	Ratio of 1:20 patients
**Counselling psychologist**	Ratio of 1:10 patients	Not specified
**Cleaner**	Ratio of 1:10 patients	Ratio of 1:10 patients
**Laundry staff**	Ratio of 1:10 patients	Ratio of 1:10 patients
**Porter**	2 per isolation centre	2 per CCU
**Epidemiologist**	2 per isolation centre	Not specified
**IPC and QA Coordinator (nurse)**	1 per 8 hours shift	Not specified
**Public health officer**	2 per isolation centre	Not specified
**Mortician**	4 per site	Not specified

In May 2020, the Kenyan Ministry of Health published interim guidelines on human resources for health (HRH) during the COVID-19 response [21], outlining the human resources needs for general isolation wards and critical care units dedicated to managing COVID-19 cases per day on average (see Table 2).

However, the document did not mention how these requirements would be attained, how staffing shortages would be addressed, nor how training needs for these healthcare workers would be met. These plans are in addition to HRH norms from 2017 for hospitals that have never been met, with a huge gap in existing skilled personnel [22]. On April 2, 2020, the government announced its plan to temporarily (3–6 months contracts) hire 6,000 health workers to help fight the coronavirus outbreak, including 5,000 for deployment to counties, and 1,000 at the national hospitals, with an aim to complete hiring by 8^th^ April 2020. Counties submitted their requirements to the national government and KSH 2.3 billion (approximately USD 21 million) of the COVID-19 support funds from national government to counties was earmarked for their recruitment [23,24]. By 21^st^ June 2020, it was reported that 4509 of the targeted 6000 had been hired [25], but there was no plan to absorb them after the contract period and many have since been laid off.

In his address on 27th July 2020, the President directed that a protocol to temporarily retain retired anaesthetists and ICU staff be developed to support the medical staff assigned to dealing with serious COVID-19 cases in the counties [26], though this appears not to have been implemented.

### 2. Training

Our interview respondents spoke to us about in-service training programmes covering different aspects of critical care and emergency care emerged over time to help bridge some of the gaps in specialist healthcare workers as illustrated in Table 3. The Emergency Triage Assessment and Treatment plus admission care (ETAT+) courses aimed at severe childhood illnesses have been running since 2006, while those run by Emergency Medicine Kenya Foundation are more recent and, in some instances, only two or three courses had been run by the time of inquiry.

**Table 3:** Prominent ongoing initiatives in Kenya towards bridging the HRH in critical care gap over the past 15yrs (as reported by respondents).

Provider	Course/Training Title	Duration	Approximate annual intake of students	Approximate total number trained
**Kenyan Paediatrics Association & Ministry of Health, Kenya**	Emergency Triage Assessment and Treatment plus admission (ETAT+) *for ages 0–5 years*	3–5 days	400	10,000
**Emergency Medicine Kenya Foundation**	‘The Emergency Care Course’ (TECC)	5 days	120	2,500
	‘Emergency Care Training for Community Health Workers’	3days	20	60
	‘Emergency Airway + Ventilation Course’	3 days	12	24
**Kenyan Society of Anaesthesiologists**	‘Safer Anaesthesia From Education (SAFE)’ for obstetrics and paediatric anaesthesia for non-physician clinician anaesthetists	3 days	One project	174
**Assist International**	Improving Perioperative and Anaesthesia Care and Training (ImPACT)	18 months	12	50

In efforts to address the HRH gap that was made worse by the pandemic, the country made efforts to scale up training for health workers. It is, however, worth noting that according to a survey from September/October 2020, while 29 (62%) of Kenya’s 47 counties had allocated a budget for capacity building, only 15 (33%) counties had developed an annual capacity building plan [27]. While certainly beneficial, the investment made in these programmes was not driven by national policy nor by a rational large-scale cost-benefit analysis of alternative approaches. Because of this, the impacts of these investments were diffuse and hard to measure or follow up, and the human resource gap remains to date.

Confidentially, our respondents reported that training was uncoordinated and non-standardised in the early days of the response, until the Ministry of Health (MoH) developed a “National IPC and Case Management Training Module of COVID-19” which is a 4-day course aimed at healthcare workers responsible for managing COVID-19 cases. This was digitised by AMREF and the MoH requested that all healthcare workers make use of the teaching materials available. The capacity building sub-committee under the National Taskforce played a role in monitoring who was trained on what and where, although key informants noted that despite the availability of a standardised national module, some partners continued to follow their own programmes. The capacity building sub-committee was working to address this and urged all counties to align their trainings with the national modules, with increasing compliance reported.

In non-governmental support, while most of the COVID-19 training targeted general case management and IPC, some stakeholders focused their efforts on supporting health care workers in managing severe or critically ill COVID-19 patients. There was however no consistent approach, and different partners linked up with different county governments to deliver diverse trainings. Gradian Health supported the Kenya Medical Training College to develop a comprehensive curriculum on “The Basics of Critical Care” to provide more guidance for nurses/medical officers who manage COVID-19 patients and staff the ICUs. It is freely available on Gradian’s online learning platform, and their stated intention is to concentrate their focus on the county-level hospitals to lead the roll-out process through simulation-based training, using existing simulation labs.

The Critical Care Society of Kenya (CCSK) also organised dedicated training sessions on the basics of intensive care and support and developed a self-teaching training package on the management of critically ill COVID-19 patients for its members [28], which had been accessed by at least 400 health care workers, mostly nurses and clinical officer and junior medical officers by the time of study. In 2020, EMKF with sponsorship from Centre for Public Health Development (CPHD) delivered their ‘Emergency Airway + Ventilation’ course to participants from three selected hospitals [29]. Assist International and Stanford University implemented a ‘Tele-mentoring Program on Oxygen Therapy and Critical Care’ in 2020, with numerous trainings made freely available online which were utilised by healthcare workers in Kenya during the pandemic. They introduced a local learning hub in Kenya from January 2020 [30].

### 3. Services and equipment available to care for the critically ill

Kenya has had several service readiness assessments/health facility assessments (HFAs) done in hospitals representing the different levels of care and including public, private, and Faith Based/NGO-based facilities from the 47 counties between 2018 and 2021. The first survey by the Emergency Medicine Kenya Foundation (EMKF) visited 186 facilities and found that 30% did not have a triage area, 60% did not have any blood bank, 80% did not have all the required guidelines (for triage, emergency care, mass casualty, and referral), and 87% could not perform all emergency procedures (including advanced critical care procedures such as resuscitation with advanced life support, rapid sequence intubation for adults and paediatric, and chest tube insertion) [31]. The second survey was done by the Ministry of Health (MoH) and included 2972 facilities [16], followed by three surveys carried out between July 2020 and February 2021 also by the MoH to assess readiness of the various facilities to provide care for patients during the COVID-19 pandemic [32,33]. The July 2020 survey assessed 1,459 facilities (239 were COVID isolation units); the December 2020 survey assessed 91 COVID facilities (74 were repeated from July); while the February 2021 survey assessed 128 facilities.

Some of the key indicators from these surveys linked to care for the critically ill are summarised in Table 4 [16,31–33]. The proportion of facilities with functioning mechanical ventilators increased from 14% of 1,459 facilities in July 2020 to 52% of the 128 facilities surveyed in February 2021. The proportion of health facilities having any oxygen source similarly increased from 58% of 1,459 facilities to 95% of 128 facilities in the latter survey. Few facilities had an oxygen plant, most relied on oxygen cylinders, and this was consistent throughout the surveys. The February 2021 report showed that only 1 in three facilities had a PSA oxygen generator or bulk liquid oxygen supply. The proportion of facilities with functioning pulse oximeters increased from 60% of 1,459 facilities in July 2020 to 90% of 128 facilities in February 2021.

**Table 4:** Summary of key indicators in service readiness assessments in Kenya between 2018–2021.

Select Indicators	EMKF HFA* 2018 [31] N = 186	KHFA** 2018/2019 [16] N = 411	Covid Readiness HFA*** July 2020 [32] N = 1459	Covid Readiness HFA Dec 2020 [32] N = 91	Covid Readiness HFA Feb 2021 [33] N = 128
Proportion of facilities with functioning ventilators (invasive and non-invasive)	22.6%	23%	14%	45%	52%
**Proportion of health facilities having oxygen (any source)**	67%	78%	58%	88%	95%
**Proportion of health facilities having an oxygen plant**	N/A	N/A	N/A	41%	38%
**Proportion of health facilities having oxygen concentrators**	N/A	N/A	47%	72%	78%
**Proportion of health facilities having oxygen cylinders**	N/A	N/A	72%	93%	91%
**Proportion of health facilities having oxygen humidifiers**	N/A	N/A	88%	91%	88%
**Proportion of health facilities having oxygen flow meters**	N/A	11%	32%	92%	96%
**Proportion of health facilities having oxygen masks**	N/A	N/A	96%	96%	97%
**Proportion of health facilities having pulse oximeters**	46.2%	58%	60%	96.7%	90%

*EMKF HFA: Emergency Medicine of Kenya Foundation Health Facility Assessment.

**KHFA: Kenya Health Facility Assessment.

***Covid Readiness MoH Health Facility Assessment.

While there was clear evidence of efforts to improve the availability of the equipment and resources required to treat very sick patients in Kenya, the situation of most hospitals was reportedly characterised by recurrent stock out of essential supplies like oxygen and damage to essential equipment. Of note was that oxygen and pulse oximeters were not consistently available. Several facilities reported their ventilators were not functional at the time of the surveys and the reasons for this included: ventilators not installed; training on their use not yet received; or funds not available for external maintenance or spare parts; and deficits in skilled personnel.

In related work, a study that modelled the hospital surge capacity of the Kenyan health system in the face of the COVID-19 pandemic in 2020 reported that, of the 64,181 hospital beds across all sectors in Kenya, only 58% have an oxygen supply; and while Kenya had 537 Intensive Care Unit (ICU) beds, it just had 256 mechanical ventilators by the time of the survey (not all were in use). Geographic disparities in hospital bed and ICU bed capacities between Kenya’s counties were also identified: just 22/47 counties reported to have had at least one ICU unit; and while 22% of Kenya’s population lived within 2 hours of a facility with an ICU available, and in 25/47 counties this was found to be 0% [12].

Despite the concentration of resources towards urban centres, ICU outcome data from Kenya suggests that even where services are available, quality of care gaps are present. One study reports an ICU mortality of 54% at Moi Teaching and Referral Hospital in Eldoret, Kenya (2014) [34], with acute respiratory failure the most common presenting diagnosis. Several challenges identified in this study include delays in transferring and appropriately triaging patients, delays in administration of antibiotics and other critical therapies, suboptimal resuscitation, and insufficient protocols to manage less critically ill patients [35].

#### Medical oxygen and ventilators

Despite recognition of the need for oxygen, by March 2021, during the third wave of the pandemic, reports emerged that health facilities were facing an oxygen crisis [36]. In early August 2020, a technical review on Oxygen supply was conducted by the MoH which resulted in several recommendations, including that the MoH should support the installation of oxygen plants in all counties; and that in more remote counties, where distribution network for cylinders was complicated by supply chain logistics and infrastructure, oxygen concentrators could be considered as an alternative to oxygen cylinders. In early January 2021, county governments were directed to enhance investments in piped and portable oxygen capacity in isolation and treatment centres during the daily press statement by the Cabinet Secretary. This appeared to be an area that has since seen considerable investment by the government [37].

Plans were also made to construct additional oxygen plants in 2021. A two-phased national oxygen roadmap was developed for the first time in Kenya [38], as a result of the pandemic, with the first phase focused on the initial response to the pandemic and prioritising 76 facilities (costed at USD 6 million) and a second phase prioritising 300 facilities (costed at USD 20 million). The status of this plan was unknown at the time of writing, however funding the plan was reportedly challenging. Promisingly, a new World Bank project was being launched with an aim to upgrade oxygen systems in 83 facilities across Kenya. Reports on these activities were not in the public domain.

Key non-governmental informants reported in the initial phase of response that they donated oxygen concentrators but that this ceased as the MoH later discouraged such support in favour of the establishment of oxygen production systems.

One example of a public-private partnership to provide medical oxygen in Kenya (Hewatele), funded through Grand Challenges Canada [39], reported receipt of public contracts worth USD 1 million to improve oxygen access at selected isolation and treatment centres. Hewatele, which operated three oxygen plants, received additional funding to hire more staff, purchase more cylinders and expand capacity for production in the face of rising demands. They planned to construct three additional plants. AMREF Health Africa commissioned construction of a medical oxygen generation plant at a Level V hospital with support from the Rockefeller Foundation. Emergency Medicine Kenya Foundation (EMKF) set up oxygen gas manifolds in the emergency departments of four county referral hospitals.

Globally, United States Agency for International Development (USAID) committed USD 18 million specifically to support the provision of medical oxygen across 11 affected countries which included Kenya, although details of their plans in Kenya were not available in the public domain [40]. Program for Appropriate Technology in Health (PATH) also supported six hospitals by assessing available respiratory equipment with a plan to support with any required repairs alongside training of biomedical engineers to maintain, to ensure sustainability over the long run. This project additionally provided pulse oximetry equipment and training and piloting of a tablet/smart-phone electronic clinical decision support algorithm for the use of pulse oximeters. Clinton Health Access Initiative (CHAI) similarly supported with pulse oximetry equipment and training to hospitals in 26 counties, through a project that pre-dates COVID-19 but has been scaled up in the pandemic. However, funding of the national oxygen roadmap that was developed in the response to the pandemic has reportedly been insufficient (anecdotal). Box 1 gives a summary of the medical oxygen market in Kenya.

**Box 1. Medical oxygen market in Kenya**

■ *Medical oxygen has been on Kenya’s Essential Medicine List since 2003*.
■ *The market for medical oxygen in Kenya has diversified in the past decade*. *Previously*, *a monopoly on medical oxygen production was held by private company BOC*, *which operates the only large liquid oxygen plant (LOX)*.
■ *Hewatele*, *a Kenyan social enterprise which pioneered a Public-Private Partnerships approach*, *broke the monopoly by constructing its first pressure swing adsorption (PSA) oxygen plant in 2014*. *It now operates 3 PSA plants*, *delivering oxygen throughout Kenya (piped and cylinder refills)*, *reportedly reducing the price oxygen price by 50 percent in Kenya*.
■ *Several PSA oxygen plants in Kenya were also built through the managed equipment services (MES) arrangement*, *a business model involving partnerships between the private sector and public healthcare providers*, *whereby government makes regular fixed payments over time for selected public hospitals to access an oxygen plant*, *instead of investing large upfront capital required to build a new plant*.
■ *As of April 2021*, *it was estimated that Kenya has 70+ oxygen PSA plants*, *although < 50% are reported functional*.
■ *Under the decentralised governance*, *counties are responsible for procuring medical oxygen*, *with differing approaches*: *some prioritise oxygen plant construction; others*, *concentrators*.
■ *Fragmented models can hamper longer-term planning to invest in larger-scale and oxygen solutions*

Beyond oxygen, Kenya’s government also procured ventilators, including 100 of Gradian Health’s Comprehensive Care Ventilators (CCVs), which have been installed in selected hospitals with associated user training targeting non-physician anaesthesia providers and 3-year service and maintenance warranties. Initial World Bank project support was linked to a plan to provide around 700 ventilators, but we were unable to verify progress with this plan. Non-governmental support in the form of medical equipment has been considerable but has perhaps lacked a shared logic across the sector. In March 2020, it was announced that the World Bank would donate 250 ventilators to Kenya, and this was followed in April 2020, by Stanbic Bank committing to donate 192 ventilators, with a further donation of an unknown number of ventilators by Jack Ma (Chinese billionaire philanthropist) that same month. USAID also donated 200 ventilators in Kenya (see S3 Table for hospital locations), from November-December 2020, and WHO reported that they also donated several ventilators. Adding these to the 100 ventilators procured by government from Gradian Health gave a total of nearly 750 new ventilators added to the estimated reported baseline of 256 ventilators available nationally in July 2020 [12]. Many of these ventilators were however not in use at the time of the study, largely due to lack of technical capacity. For example, a hospital in Nairobi reportedly had 27 ventilators, of which 18 donated by World Bank, five donated by USAID and four initially procured by the hospital, but the hospital had only one anaesthesiologist and few critical care nurses, which restricted the use of the 27 ventilators [24].

While these investments in ventilators have potential for long-term impact, this is clearly contingent on the nature of support provided to make good use of this equipment. Gradian Health, for example, provides a comprehensive three-year warranty cycle throughout which they support with training, technical and maintenance support and maintain active communication and follow-up with their users: an engagement model that does not just end with the machine. The extent to which other equipment donations include such extensive support is unknown and likely varies, however several key informants expressed concern that absorption capacity for advanced equipment is heterogenous across hospitals and that some were not able to utilise these valuable contributions as intended, rendering them wasteful. It was noted that some hospitals received ventilators, despite not having any access to piped oxygen, which calls into question the appropriateness of resource allocation.

Little information was shared regarding non-invasive ventilation equipment support, including continuous positive airway pressure (CPAP) and bi-level (BiPAP), suggesting that this area was not heavily prioritised by external stakeholders supporting Kenya’s COVID-19 response, despite rapidly emerging evidence of the value of this approach and greater feasibility compared to invasive ventilation [41].

### 4. Policy efforts to strengthen care for the critically ill

Targeting personnel to care for critically ill, The Kenya Society of Anaesthesiologists (KSA) reported having a strategic plan spanning 2020–2024, with a target to increase the anaesthetist to patient ratios to 3:100,000 by 2023, by increasing the number of training accredited sites and establishing new cadres of practice [42]. For infrastructure, Kenya has a Health Infrastructure Norms and Standards guidance (2017) in which the standards for ICU and HDU bed capacity are defined as 12 beds each for secondary level care hospitals (in Tier 3, see Fig 1), and 24 beds each for national tertiary referral hospitals (in Tier 4) with recommendations for equipment detailed [43]. The country recently launched a comprehensive National Emergency Medicine Care Policy and Strategy (spanning 2020–2025), aiming to address current gaps in emergency medical care funding, management, workforce, and infrastructure. At the time of writing there did not exist a single, consolidated strategy or policy document covering critical and emergency care. It is of considerable note however, that the Ministry of Health with support from the Emergency Medicine Foundation of Kenya developed The Kenya Emergency Medical Care (EMC) Policy 2020–2030 –the first-ever policy in Kenya that seeks to establish a working Emergency Medical Care (EMC) System as a key component of the healthcare system in the country [44].

#### National-level pandemic response

From the start, the COVID-19 response in Kenya was led by the highest levels of the government which helped mobilise action across different levels and sectors. Even before the first case of COVID-19 was confirmed in Kenya on 13 March 2020, the Kenyan MoH had developed an early contingency plan spanning February to April 2020, budgeted at approximately USD 7,545,000, with a focus on preparedness and early response to the outbreak. This plan aligned with the key actions prioritized in the WHO draft Operational Planning Guidelines to Support Country Preparedness and Response [45], and identified 14 initial priority counties for the response [46]. With the support of the National Taskforce and its sub-committees, the Kenyan MoH has developed numerous documents to guide the health response, including strategies, guidelines, circulars and SOPs as indicated in S2 Table Summary of Summary of COVID-19 documents produced by Kenyan Ministry of Health.

**Fig 3:**
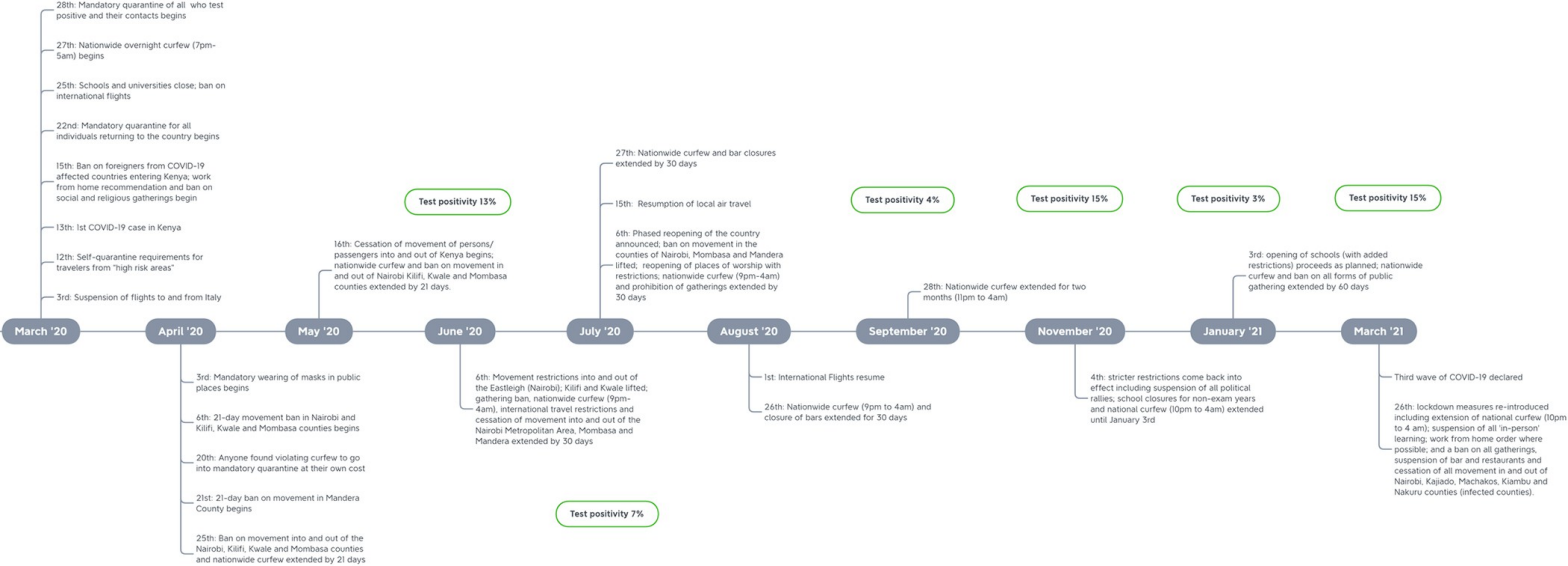
Timeline of Key policy events during the study period.

Fig 3 shows a summary of key policy events linked to the pandemic response.

#### Government policy, guidance and support for critically ill COVID-19 patients

Bolstering the capacity of hospitals to manage critical patients was an early stated aim of the government. This was largely based on assessments of capacities that showed gaps in ICU beds across counties; and on projected caseloads that were widely expected to place undue strain on hospitals’ inpatient admissions. One early modelling study predicted that Kenya would need an additional 1,511 ICU beds and 1,609 ventilators to absorb COVID-19 cases under a scenario of the transmission curve being concentrated over 6-months, reducing to 374 and 472 ICU beds under a scenario where the transmission curve flattened to 18 months and 12 months, respectively [12]. There were concerns that counties would not be able to handle severe or critical COVID-19 patients and that hospitals in Nairobi would become overwhelmed with referrals from across the country, which actually happened in the earlier waves, before the counties got better equipped.

#### Policies on identification and movement of COVID 19 patients

The guidelines for clinical management of severe and critical COVID-19 patients are described in the Interim Guidelines on Management of COVID-19 in Kenya [47] and in the Guidelines on the management of paediatric patients during COVID-19 pandemic [48], both of which were developed and disseminated in March 2020. According to this plan, suspected patients would call a national Call Centre to trigger a visit by Fast Response Teams and, for patients requiring hospitalisation, be transported by emergency vehicle or safely transport themselves to the appropriate health facility for management. Upon arrival at the hospital, all patients would be triaged to identify any patients with respiratory and other severe manifestations of disease (according to defined clinical syndromes), for immediate supportive care and treatment. In line with this, all health facilities were advised to set up triage stations near the entrance to the facility, and to identify a room for isolation of suspected patients awaiting transfer [47].

#### County-level pandemic response

While continuing to lead on policy, strategy and communication, the national government made its 47 counties accountable to prepare for and respond to COVID-19, consistent with the devolved governance principles. To this end, county governors formed independent response coordinating committees to manage the pandemic at the county level, in line with the national guidance. An estimated USD 63.8 million were disbursed by the national government to all counties in June 2020, to bolster health facilities’ readiness [49]. In addition, counties were expected to draw on their own financial mechanisms and resources, to supplement the funds received.

### 5. Funding and other modalities of support in COVID-19 health response

Government mobilisation and distribution of support took different forms. By the end of 2020, Kenya had reportedly spent approximately USD 2.4 billion on the overall COVID-19 response, which represented 2.4% of the GDP, the second-highest budget amongst low middle-income earning countries [50]. For health activities specifically, an estimated USD 218.3 million was allocated to the Ministry of Health for the pandemic, of which approximately USD 155.8 million was from the exchequer and USD 62.5 million from development partners. In Kenya, health expenditure at the hospital level is managed through county governments and much of the funding allocated to health was channelled through these structures.

The second modality was increased funding to partner agencies, particularly those with prior relevant expertise before the pandemic. This type of support, to a large extent, depended on donor interests and priorities, as well as agencies existing skills, knowledge and track-record in a given area of work.

Overall, as of March 2021, we determined that partner agencies had received approximately USD26.2 million funding for health activities (summarised in Table 5 below), although this excluded the value of in-kind donations and any intersectoral funding, and likely under-estimates the true amount.

**Table 5:** Summary of information found on new funds to partner agencies to support Kenya’s COVID-19 response.

Funder/Donor	Agency	Amount USD (million)
**European Commission’s Humanitarian Aid and Civil Protection Department**	WHO	3.367
**JICA**	UNICEF & UNDP	3.115
**Canada, Government of**	WHO	1.123
**Germany, Government of**	WHO	0.2
**Japan, Government of**	UNICEF	0.857
**DFID**	KEMRI	0.786
**United Kingdom, Government of**	WHO	0.0713
**King Baudouin Foundation.**	WHO	0.202
**Germany, Government of**	WHO	0.9
**Bill and Melinda Gates Foundation**	WHO	0.15
**UN Women**	UNFPA	0.014
**Multiple**	CPHD	0.3
**The Global Fund**	AMREF	9.0
**The Global Fund**	KRC	6.1
**TOTAL**		**26.1853**

**Fig 4:**
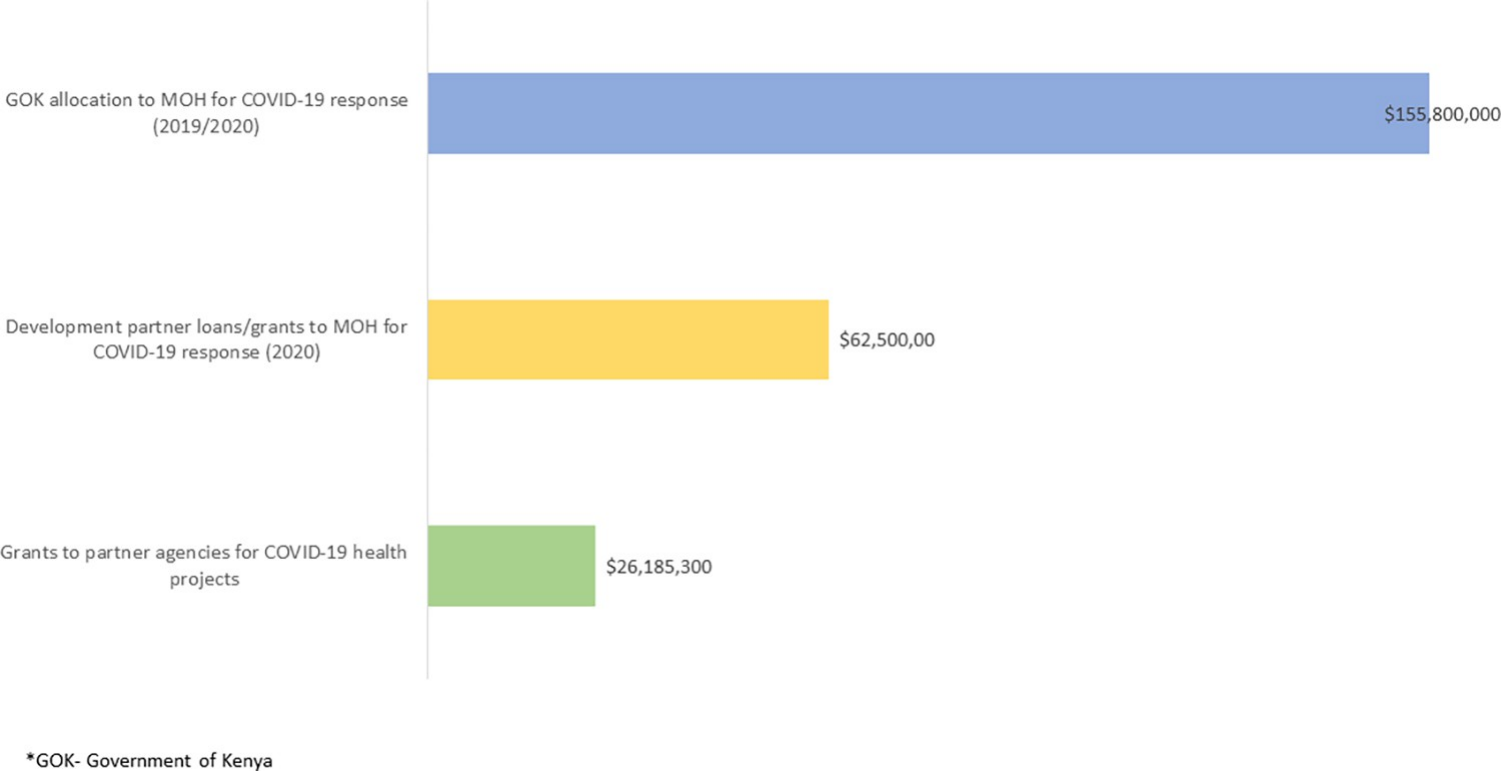
Scale of funding to the MoH vs implementing partners for the COVID-19 response in Kenya.

In general, however, additional new funding received by partners specifically for COVID-19 health activities was reported by key informants as quite minimal or not forthcoming as compared to direct government support (see Fig 4), and the need for more proactive resource mobilisation was reported. Indeed, the 2020 humanitarian flash appeal for the health response to this crisis was funded at just 15% of the USD 56.5 million appeal based on information reported by participating organisations [51].

To address the general lack of funds, partner agencies identified further support by repurposing funds for existing programmes. Examples included UK Development For International Development (DFID)-funding to UNICEF nutrition programming which was redirected to support messaging around COVID; KEMRI-Wellcome Trust repurposed some of their study teams to COVID-19 surveillance projects; and PATH repurposed one of their projects to train county staff on COVID-19 management and to assess available respiratory equipment and support with repairs. Such arrangements appeared to be shorter-term adjustments rather than reflecting changes to broader programme strategies but were appreciated by key informants and allowed for rapid implementation of urgent activities.

In-kind donations of consumables and equipment were another prominent form of support from private sector, faith-based organisations and other donors. The donations included ventilators, personal protective equipment (PPE), laboratory supplies, hand sanitisers among others. This support was criticised by some respondents as an opportunity for high-level visibility by donors, without due consideration to the need for and appropriateness of the materials and equipment. For some equipment, such as ventilators, donations surpassed the requirement and capacity to use in some cases. On the other hand, respondents also described how many such donations, particularly of consumables which were in short supply such as laboratory reagents or PPE, played an important role. Such donations also likely helped to rapidly offset some of the costs faced by Kenyan hospitals in providing care for severe COVID-19, estimated at USD 119/patient/day in general hospital wards and USD 562/patient/day in intensive care units [52].

Finally, high performing facilities in the faith based and private sector became important actors in delivering high-quality care. These hospitals had over time accrued expertise that allowed them to offer substantially higher-level provision of care for the critically ill that was useful during the pandemic. At Kijabe Mission Hospital, efforts were greatly strengthened by their Emergency and Critical Care Clinical Officer (non-physician) graduates who were able to comfortably manage all the intubation/ventilation procedures with little additional training support beyond COVID-19 basics. Similarly, the Aga Khan University Hospital, was very proactive in re-organising to meet COVID-19 needs, developing trainings and protocols. While such facilities are atypical in many regards, they did contribute significantly in managing COVID-19 caseloads in Kenya and, to some extent, relieved the burden on the public sector for patients who could afford their services (ICUs in private facilities reportedly required a deposit of between 1,500–6,000USD at the height of the pandemic) [53].

## Discussion

In reviewing Kenya’s response to the pandemic, we are able both to offer a historical overview of COVID-19 in an important and influential African state, and to point to constructive insights into how countries might prepare for future emergencies. Our findings reveal a strong central capacity to set guidelines and policy, but a health system without sufficient capacity to deliver essential and emergency care at scale. Although plans were quickly forged, they were not built on the real-world capacities of Kenya’s health facilities and workforce, and we stress the need to make general health systems strengthening a part of future emergency planning.

Support to Kenya’s pandemic response could broadly be categorised into different modalities. The first and most important modality was in direct government support. The Government of Kenya’s (GoK) COVID-19 response was the clear leader of the policy and direction in the country; it was the main conduit for funding; and the most important single actor. Estimating the proportion of investments that were allocated to strengthen support for critically ill COVID-19 patients for non-governmental actors was not possible due to a scarcity of detailed information. However, findings from the key informant discussions suggest that the non-governmental direct financial response could have been more substantial. Explanations for this varied, with some suggesting that strengthening capacity to care for the critically ill required longer-term investments and planning to address, for example, the insufficiency of specialised human resource and to commission new infrastructure such as oxygen productions systems. This was further complicated by the need for high levels of technical expertise and costly investments. In addition, it was noted that donors were not typically familiar with differences in levels of clinical care and may not be well informed about how to support curative services, as compared to preventive measures. Despite these concerns, there were some notable examples of external investments and support specifically directed at strengthening care for the critically ill, particularly in relation to medical oxygen, medical equipment, and training.

Our findings suggest that the pre-pandemic health structure and resources available for the treatment of the critically ill in Kenya were sparse and insufficient and were not able to meet pre-pandemic and pandemic emergency and critical care demand. There were major limitations in human resources, equipment and facilities and where critical care services were notionally available, they lacked key material and skilled personnel. For many Kenyans, critical care was simply not accessible. The pandemic response galvanised action by the Government of Kenya and various other non-governmental agencies to mobilise resources and carry out activities to mitigate the effects of the pandemic. The bulk of the efforts were initially however largely directed towards more advanced critical care resources like ICUs and ventilators, and since not much could be done to match the human resource gap in a short time, a lot of the advanced technology equipment remained unused. These efforts later progressed to scaling up oxygen.

We also observed that despite strong policy and appropriate guidance on what resources should be available at each level of facility in Kenya, the reality on the ground was that many of these ‘required’ resources were lacking. We believe this demonstrates the need for a holistic and evidenced-based approach to developing capacity in Kenya’s hospitals, especially in primary and secondary level care (Tier 2–3) facilities. Given the known pre-existing shortages in specialist human resources compounded by national strikes among doctors, nurses, and clinical officers’ unions due to poor COVID-19 related working conditions from November 2020-February 2021, staffing has remained a limiting factor in scaling up capacity of hospitals to care for critically ill patients despite the government’s efforts and investments in this regard.

The lack of human resource is fundamental and the consequences of the lack of skilled personnel was exposed during the various waves of the pandemic. As a result of shortages in trained specialists, there has been a strong reliance on generalist (non-specialist) and junior doctors, non-physician clinical officers and nurses to manage critically ill patients, under the supervision of the very few on call-specialists, as is the case in many other resource-limited setting [54]. We suggest that planning for the treatment of the critically ill in Kenya should prioritise the human resources required to provide the most effective forms of support to the widest group of patients. Though plans are in place to expand the number of anaesthetists in Kenya, we recognise the significant costs and time associated with training health workers to this level, as well as the challenge of recruitment and retention in rural areas, and believe the government could prioritise a public health approach focussing on essential emergency and critical care (EECC) at scale using lower cadre staff with a potential to save more lives at lower costs [5,55].

As the pandemic began, the primacy of the national government in all matters was clear. There is evidence that strong central government leadership in the pandemic helped counties with target setting and that hospitals were to some extent engaged in this endeavour through county governance structures. Given the centrality of government in this space, there is need to identify the best ‘entry point’ for engaging hospital management, which will be essential in planning any future initiatives, within the devolved governance structures. The central government both received, and allocated from its own budget, the most substantial investments in critical care for COVID patients. For health activities specifically, an estimated USD 218.3 million was allocated to the Ministry of Health for the pandemic, of which approximately USD 155.8 million was from the exchequer and USD 62.5 million from development partners. Additionally, from the earliest days of the pandemic, they produced sensible and timely guidance and reporting through a range of diverse and capable working groups.

This centralised policy system however was not able to prevent the potential wastage of funds on high-priced critical care equipment that is unlikely to be effectively deployed, however. In much the same way as has been noted for higher-income settings, spending on vastly increasing the number of ventilators was misguided. Although upwards of 750 ventilators were donated, Kenya had only 234 critical care nurses and only 202 physician anaesthetists for the population of approximately 47 million. These numbers are far fewer than the 1:6 ratio of anaesthetists to patients and 1:1 ratio of nurses to patients who would be required to make use of them of the ventilators. Relatedly, though the central government was able to raise significant funds to support the response, there were concerns regarding how much of the sums raised got deployed into improving care, with widespread reports of alleged corruption and wasted opportunities. Despite this, much positive activity was undertaken and the wealth of actors working in different fields represents a further opportunity for a greater united response oriented around the government’s highly effective policy-making apparatus.

We noted a lack of a pre-pandemic consolidated strategic plan to strengthen care for the critically ill. The pandemic led to increased interest in this domain. There is a clear ‘appetite’ for such a plan, and with it, the potential to explore EECC implementation modalities. There is need to build on, or connect with, other existing initiatives such as the new Emergency Care Policy by the Ministry of health in conjunction with Emergency Medicine Kenya Foundation. [17]. Strengthening care for the critically ill was perceived as a highly technical area which can be difficult for donors to engage with, and “care of critical illness” is often conflated by stakeholders with “intensive care” or “emergency care”. Instead, most critically ill patients are cared for in general wards [6]. A clear strategic plan containing a focus on essential care for all critically ill patients everywhere and not just in large tertiary care settings would help bridge the gap. Kenya has a successful track record of embedding/institutionalising new initiatives such as ETAT+ (noting that this took 10–15 years) and mobilising resources around this; from which lessons can be drawn [56–58].

### Study strengths and limitations

Whilst these were difficult times to engage in research in Kenya and worldwide (with very real constraints on data collection) the broad range of sources included in our desk review and across our stakeholder group enabled us we believe to produce findings that these stakeholders found credible. The diverse group of stakeholders identified for this study enabled depth of understanding concerning the funding and resources potentially available to strengthen critical care in Kenya although detailed information on non-governmental funding, actual funds disbursement and scale of implementation activities is sometimes lacking. However, we used discussions with important stakeholders to triangulate findings from the desk reviews. We also fed-back findings to participants and to the Kenya MoH helping to validate our findings. We noted considerable goodwill among practitioners and other stakeholders to improve access to quality care for the critically ill in all hospitals given the wider benefits beyond COVID-19. There is interest in using this window of opportunity to drive change.

## Conclusions

While emergency response mechanisms are not conducive to addressing longer-term health system issues, the pandemic increased global recognition of the need to fund health systems approaches to address care for the severely ill. Despite difficulties encountered with conducting research in Kenya during the pandemic, considerable interest and motivation was observed among stakeholders to improve service availability and quality of care for the critically ill in hospitals, given the broad recognition of the benefits of increased capacity beyond COVID-19. This, combined with renewed global interest and funding, presents an opportunity for the introduction of the concept of Essential Emergency and Critical Care (EECC) in LMIC settings, as part of broader national strategies to improve access to essential care for the critically ill. Implementation modalities in LMIC settings will require close consultation with pre-existing stakeholders and may benefit from the formation of a network of experts from different clinical fields under the leadership of respective ministries of health to reduce silos, optimise use of technical expertise and other resources, and to ensure alignment with ongoing initiatives. This could enable implementation in general wards and in secondary level care facilities, where most of the critically ill patients are likely cared for and result in increased survival at scale. Box 2 gives a summary of the policy insights and recommendations of this work.

**Box 2 Policy insights and recommendations**

***Low capacity:** Kenya, like many LMICs, has a low capacity to deliver general hospital care and a much lower capacity for ICU-based care. While there are major limitations in appropriate equipment and facilities, there is a significant shortfall of skilled human resources to support critical care. Planning should prioritise human resources to provide the most effective support to the widest group of patients focussing on principles of essential emergency and critical care (EECC)*.

***Primacy of central government:** the Kenyan government reacted quickly and created sensible guidelines and policies throughout the pandemic, drawing on local and international expertise. How policies were translated into practice was less clear. Need for a clear strategic plan with a focus on essential care for critically ill patients at all levels of care building on existing initiatives with Emergency Medicine Kenya Foundation*.

***Funding:** The pandemic resulted in large outlays of funding to support the response, but this funding was not matched to specific, evidence-based strategies and instead, focusing on capital purchases such as ventilators that could not be usefully deployed. Efforts could be better directed towards more basic but essential supplies like oxygen*.

***Gaps in critical care policy and practice**: Policies that will guide future development of emergency and critical care that do not require intensive care units and instead optimise for cost and impact and a public health approach using EECC principles*.

## Supporting information

S1 Checklist Inclusivity in global research checklist. (DOCX)

S1 Fig The Essential Emergency and Critical Care (EECC) framework [7,8]. (DOCX)

S1 Table Details of key stakeholder discussions. (DOCX)

S2 Table Summary of COVID-19 documents produced by Kenyan Ministry of Health. (DOCX)

S3 Table Location of USAID donated ventilators in Kenya. (DOCX)
