# Supplementary figures and images for: Policies and resources for strengthening of emergency and critical care services in the context of the global COVID-19 pandemic in Kenya
Source: PLOS Glob Public Health. 2023 Jul 3;3(7):e0000483. doi: 10.1371/journal.pgph.0000483 (PMC10317215; doi:10.1371/journal.pgph.0000483)

### S1 Fig: The Essential Emergency and Critical Care (EECC) framework


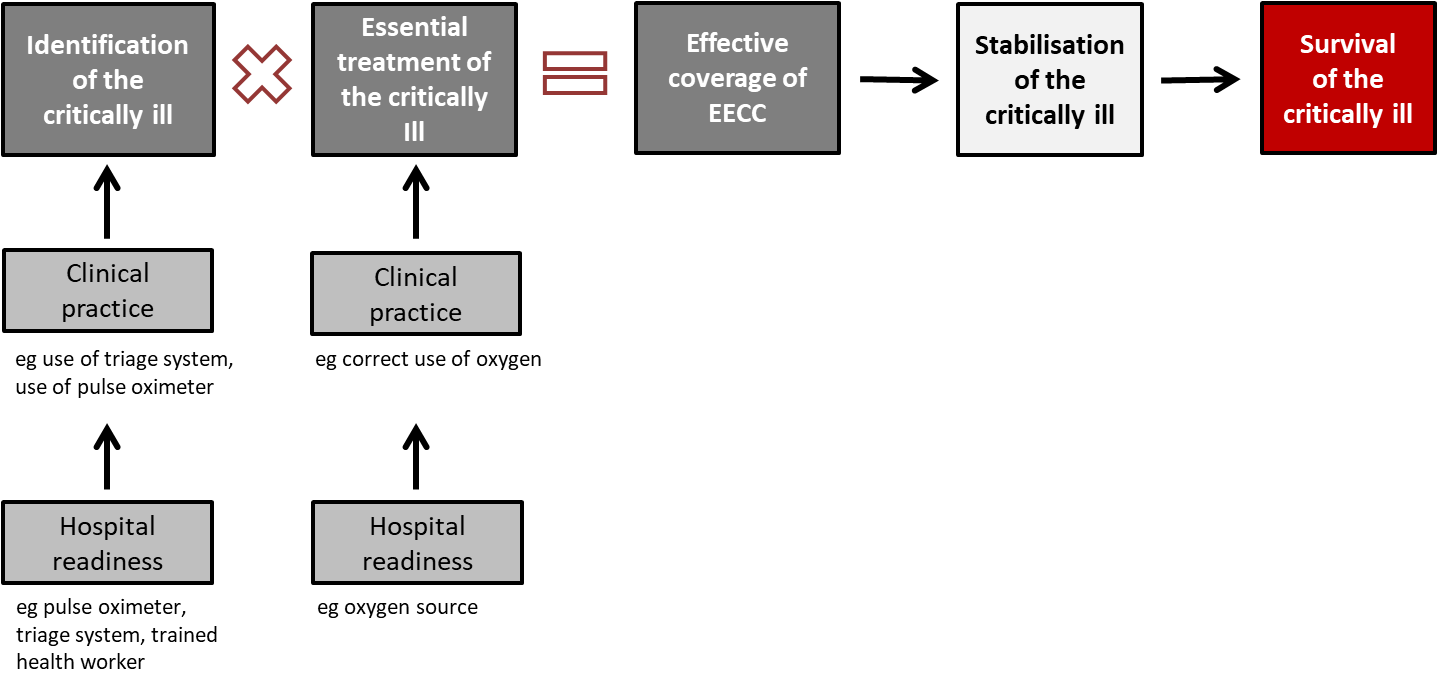

Supplement: S1 Fig — (DOCX) [file pgph.0000483.s002.docx]
