# Supplementary material for: Policies and resources for strengthening of emergency and critical care services in the context of the global COVID-19 pandemic in Kenya
Source: PLOS Glob Public Health. 2023 Jul 3;3(7):e0000483. doi: 10.1371/journal.pgph.0000483 (PMC10317215; doi:10.1371/journal.pgph.0000483)
Supplement: S1 Table — (DOCX) [file pgph.0000483.s003.docx]

### S1 Table: Details of stakeholder key information discussions and interviews

| **S/N** | **Agency/organisation** | **Setting** | **Date of discussion/interview** |
| --- | --- | --- | --- |
| 1 | Gradian Health | Global | 19/01/2021 |
| 2 | World Federation of Societies of Anaesthesiologists (WFSA) | Global | 25/02/2021 |
| 3 | Emergency Medicine Kenya Foundation | Kenya | 25/11/2020 |
| 4 | Aga Khan University Hospital | Kenya | 25/11/2020 |
| 5 | Christian Health Association of Kenya (CHAK) | Kenya | 15/12/2020 |
| 6 | Centre for Public Health and Development (CPHD)/Hewatele | Kenya | 12/01/2021 |
| 7 | KEMRI Wellcome | Kenya | 13/02/2021; 20/01/2021 |
| 8 | PATH | Kenya | 29/01/2021 |
| 9 | DFID | Kenya | 01/02/2021 |
| 10 | WHO | Kenya | 03/02/2021 |
| 11 | Critical Care Society of Kenya (CCSK) | Kenya | 03/02/2021 |
| 12 | Ministry of Health | Kenya | 05/03/2021 |
| 13 | The Global Fund | Kenya | 12/03/2021 |
| 14 | African Mission Healthcare | Regional | 10/12/2020 |
| 15 | AMREF | Regional | 28/01/2021 |
