# Supplementary material for: Policies and resources for strengthening of emergency and critical care services in the context of the global COVID-19 pandemic in Kenya
Source: PLOS Glob Public Health. 2023 Jul 3;3(7):e0000483. doi: 10.1371/journal.pgph.0000483 (PMC10317215; doi:10.1371/journal.pgph.0000483)
Supplement: S2 Table — (DOCX) [file pgph.0000483.s004.docx]

### S2 Table Summary of COVID-19 documents produced by Kenyan Ministry of Health

| **Month** | **Strategies, guidelines, circulars and SOPs issued by MoH** |
| --- | --- |
| January 2020 | National 2019 novel coronavirus Contingency (readiness and early Response) plan |
| March 2020 | Interim Guidelines on Management of COVID-19 in Kenya |
|  | Guidelines on the management of paediatric patients during COVID-19 pandemic |
|  | COVID-19 Quarantine protocols |
|  | COVID-19 guidance on comprehensive HIV service delivery |
|  | Circular on suspension of elective surgical procedures |
|  | Case definitions for COVID-19 |
|  | Circular on treatment of TB patients during COVID-19 pandemic |
|  | Guidance for nutrition management of COVID-19 for health workers in COVID-19 treatment and isolation centres |
|  | Guidelines for dental practice with regards to the COVID-19 pandemic |
|  | Interim guidance for implementing home care of people not requiring hospitalization for corona virus disease (COVID-19) |
|  | Literacy material for people living with HIV |
|  | Occupational safety and health advisory on coronavirus (COVID-19) |
|  | Public mental health education awareness |
|  | Guidance for infection prevention and control for coronavirus disease (COVID-19) in homes and residential communities |
| April 2020 | Interim guidance for public use of face masks to reduce droplet transmission for COVID 19 |
|  | Interim guidelines on handling of human remains infected with COVID- 19 in Kenya |
|  | Key nutrition messages in prevention of COVID-19 |
|  | Circular on NCD clinics during COVID-19 outbreak |
|  | Practical Guide for Continuity of Reproductive, Maternal, Newborn and Family Planning Care and Services in the Background of COVID19 Pandemic |
|  | Standard operating procedure in handling of health record and information management during the COVID-19 pandemic |
|  | Guidelines on Continued Provision of Community Health Services in the Context of Corona Virus Pandemic in Kenya |
|  | Interim Guidance for Nutrition and Dietetics Management of COVID-19 for Health Workers in Treatment and Isolation Centres |
|  | Interim Guidance on Continuity of Nutrition Services Delivery in the Context of COVID-19 Pandemic |
|  | Physiotherapy guidelines on management and recommendations to clinical practice for COVID-19 |
|  | Standard operating procedures (SOPs) for pre and post-test counselling- MHPSS providers |
|  | Psychological first aid (PFA) guide for COVID-19 response in Kenya |
|  | A comprehensive guide on mental health and psychosocial support during the COVID-19 pandemic |
| May 2020 | Kenya COVID-19 emergency response project labour management procedures |
|  | Interim guidelines on human resources for health (HRH) during COVID-19 response |
|  | Guidance on the rationale use of PPE for COVID-19 in healthcare settings |
| June 2020 | Home based isolation and care guidelines for patients with COVID-19 |
|  | Division of National Malaria Program plans to continue with essential services in the wake of COVID- 19 |
|  | Interim guidance for health and safety measures in workplaces in the context of COVID-19 |
|  | Protocol for management of restaurants and eateries during the period of COVID-19 pandemic |
|  | Guidelines for food business operators (FBOs) on re-opening of restaurants and eateries during COVID-19 pandemic |
| July 2020 | Interim Guidance on Provision of Services for Non-communicable Diseases (NCDs) During the COVID-19 Pandemic |
|  | Targeted testing strategy for Coronavirus disease 2019 (COVID-19) in Kenya |
|  | Interim Guidance on Continuity of Trauma Care Services During COVID 19 Pandemic |
| August 2020 | Environmental and social management framework for Kenya COVID-19 emergency response project |
|  | Infection control and waste management plan (ICWMP) for Kenya COVID-19 emergency response project |
| September 2020 | Revised Interim guidelines on handling decedents suspected or confirmed for COVID-19 |
| October 2020 | Guidelines to be used by Occupational Therapists in rehabilitation of Patients during COVID-19 Pandemic |
| December 2020 | COVID-19 Antigen Rapid Diagnostic Testing: Interim Guide |
| January 2021 | Interim guidance for blood management intra COVID-19 |
|  | Guide on digital verification of COVID-19 certificates |
|  | Travel guide and Traveller manual |

*These policy documents were obtained from the Ministry of Health Kenya, and those in the public domain can be found here <https://www.health.go.ke/#1621662557097-37ed30fd-e577>
