# Supplementary material for: Policies and resources for strengthening of emergency and critical care services in the context of the global COVID-19 pandemic in Kenya
Source: PLOS Glob Public Health. 2023 Jul 3;3(7):e0000483. doi: 10.1371/journal.pgph.0000483 (PMC10317215; doi:10.1371/journal.pgph.0000483)
Supplement: S3 Table — (DOCX) [file pgph.0000483.s005.docx]

### S3 Table: Location of USAID donated ventilators in Kenya

| **Location** | **Number** | **Timeframe** |
| --- | --- | --- |
| Mombasa County Coast General Teaching and Referral Hospital | 14 | 02 November 2020 |
| Kiambu County- Kijabe mission hospital (7), Thika Level Five (4) and Tigoni Covid-19 center (9) | 20 | 14 December 2002 |
| Meru County Hospital | 7 | 16 December 2020 |
| Embu County Hospital | 10 | 16 December 2020 |
| Muranga County Level 5 Hospital | 10 | 16 December 2020 |
| Nyeri County- County Hospital and Kenyatta National Hospital-Othaya Annex | 14 | 17 December 2020 |
| Isiolo County Teaching and Referral Hospital (ICTRH) | 4 | 17 December 2020 |
| Busia County Referral Hospital | 4 | 17 December 2020 |
| Nakuru County Government’s Level V Hospital | 10 | 17 December 2020 |
| Kakamega County General and Referral Hospital | 10 | 18 December 2020 |
| Uasin Gishu County Moi Teaching and Referral Hospital MTRH | 20 | 20 December 2020 |
| Migori County Referral hospital | 4 | 21 December 2020 |
| Nairobi County Kenyatta National Hospital as well as KNH IDU Mbagathi and KNH Othaya | 36 | 22 December 2020 |
| Kisumu County Jaramogi Oginga Odinga Teaching and Referral Hospital | 10 | 22 December 2020 |
| Kwale County | unknown | 23 December 2020 |
| Kajiado County Referral Hospital | 4 | 23 December 2020 |
| Garissa County Referral Hospital | 4 | 24 December 2020 |
| Nairobi County Mama Lucy Kibaki Hospital | 5 | 29 December 2020 |
| **TOTAL** | 186/200 |  |
